# Evaluating the effect of the mitochondrial alternative peptide MTALTND4 on gene expression

**DOI:** 10.1016/j.bbrep.2025.102223

**Published:** 2025-08-27

**Authors:** Ludovic Nadeau-Lachance, Thierry Choquette, Hajar Hosseini Khorami, Annie Angers, Sophie Breton

**Affiliations:** Département de Sciences Biologiques, Université de Montréal, Montréal, Québec, Canada

**Keywords:** Mitochondria, Mitochondria-derived peptides, Alternative proteins, Small open reading frames

## Abstract

• This is a preliminary study exploring the effect of the mitochondrial alternative peptide MTALTND4 on gene expression in two different culture media using microarrays, RNA-seq and RT-qPCR.

• Microarrays in MiR05 medium suggest that exogenous treatment with MTALTND4 may alter gene expression and that responsive genes are mostly related to cell metabolism.

• Conversely, RNA-seq in DMEM low glucose suggests that MTALTND4 has a negligible impact on gene expression.

• RT-qPCR in MiR05 or DMEM low glucose indicates that the culture media affects gene regulation.

• This study emphasizes the importance of employing diverse approaches when examining the physiological effects on cells and the need to carefully select the appropriate culture media to interpret cellular responses and expression data accurately.

## Highlights

• This is a preliminary study exploring the effect of the mitochondrial alternative peptide MTALTND4 on gene expression using microarrays, RNA-seq and RT-qPCR.
• Microarrays and RT-qPCR suggest that exogenous treatment with MTALTND4 may alter expression of genes mostly related to cell metabolism.
• Conversely, RNA-seq suggests that MTALTND4 has a negligible impact on gene expression.
• The culture media also affects gene regulation.
• This study emphasizes the importance of employing diverse approaches when examining the physiological effects on cells.
• It also emphasizes the need to carefully select the appropriate culture media to interpret cellular responses and expression data accurately.

## 1 Introduction

The coding potential of the human mitogenome has proven to be far more important than previously recognized in recent years, with functional open reading frames (ORFs) identified within annotated mtDNA regions [1]. A first class of small ORFs encoding mitochondria-derived microproteins (MDPs of 16–38 amino acids [AA]) has been confirmed within mitochondrial ribosomal genes, including Humanin, small humanin-like peptides (SHLPs 1–6) and MOTS-c [[1], [2], [3]]. These MDPs are translated in the mitochondria or cytoplasm and are involved, among other things, in mitochondrial function, cell proliferation, apoptosis, and cytoprotection [1].

A second class of mitochondrial ORFs, overlapping or nested within protein-coding regions, has recently been identified. These include SHMOOSE (58AA) [4] and MTALTND4 (99 AA) [5] in 2023, followed by CYT-187AA [6] and MTALTCO1 (259 AA) (Robitaille et al., 2024 *BioRxiv*) in 2024. Notably, MTALTND4 was the first ORF fully embedded in a different (alternative) reading frame of a protein-coding gene (ND4) [5]. Like other MDPs, MTALTND4 modulates cellular bioenergetics. Specifically, it reduces mitochondrial respiration and cell proliferation without affecting viability [5]. MTALTND4 also interact with the protein C1QBP, which play a key role in mitochondrial gene regulation and cellular energy metabolism [5]. These results prompted us to investigate whether it could also modulate nuclear gene expression, as has been reported for other MDPs like Humanin and MOTS-c [1].

The primary aim of this exploratory study was therefore to assess whether MTALTND4 affects gene expression. We began by applying the same microarray approach used for MOTS-c (HeLa and HEK293T cells exposed to 10 μM synthetic peptide for 4 h) but in MiR05 medium, i.e. the same medium used in our physiological study [5], to facilitate comparison with prior functional results. We then sought to replicate and extend these findings using RNA sequencing (RNA-seq), anticipating that RNA-seq might reveal a broader set of differentially expressed genes (DEGs) due to its greater sensitivity. For the RNA-seq experiments, cells were exposed to 10 μM synthetic peptide for 4 h in DMEM low glucose (MiR05 being unsuitable for long-term culture) and peptide exposure was also extended to 72 h. RT-qPCR was used to validate key findings for both cell culture media.

We expected overlapping DEGs between the microarray and RNA-seq platforms. Instead, we observed minimal overlap and, in the case of RNA-seq, very few DEGs overall. We also expected to validate some of our microarray results in the MiR05 medium and RNA-seq findings in the DMEM medium with RT-qPCR, but no significant changes were observed in the validation of top DEGs. This approach, however, highlighted the potential influence of culture medium on DEG detection. Our study emphasizes the importance of considering the potential variability introduced by method and culture conditions. It underscores the need for standardized protocols and multi-platform validation to ensure reliable transcriptomic findings.

## 2 Materials and methods

Microarrays were performed as described in Ref. [3] for MOTS-c, but with the cells suspended in MiR05, a medium that supports mitochondrial function while preventing the inhibition of mitochondrial processes that could introduce bias into the data [7]. Specifically, HEK-293T and HeLa cells were cultured at 37 °C with 5 % CO_2_ in Dulbecco's Modified Eagle Medium (DMEM), supplemented with 10 % Calf Bovine Serum, 1 % penicillin-streptomycin antibiotics, and 1 % fungizone antifungal, then transferred to MiR05 (110 mM d-sucrose, 60 mM lactobionic acid, 20 mM taurine, 20 mM HEPES, 10 mM KH_2_PO_4_, 3 mM MgCl_2_, 0.5 mM EGTA, BSA 1 g∙L-1; supplemented with 5 mM pyruvate). Approximately 25 000 cells were seeded into individual wells of 24-well plates with MiR05 and treated exogenously with either 10 μM synthetic MTALTND4 (LifeTein, Somerset, NJ, USA) or water for 4 h, with each condition replicated two times (n = 2 for each condition). Cells were then pelleted by centrifugation and frozen at −80 °C. Total RNA of treated and control cells was extracted using the Quick-RNA™ Miniprep kit (Zymo Research Corporation, Irvine, CA, USA) following the manufacturer's protocol. RNA samples were evaluated for both quantity and purity by electrophoresis on a 1 % agarose gel and spectrophotometry using a BioDrop μLITE spectrophotometer. Samples were then sent to Génome Québec (Montréal, QC, CA) for a Human Clariom S Assay via ThermoFisher Scientific (Affymetrix Inc., Santa Clara, CA, USA). Microarray data was interpreted with ThermoFisher's Transcriptome Analysis Console (TAC) Software version 4.0.2.15 (Affymetrix, Inc.): the data were normalized by the robust microarray averaging (RMA) method and differentially expressed genes were chosen through false-discovery rate <0.30, fold-change >1.5 in both directions and an ANOVA p-value <0.05, as in Ref. [3]. Gene ontology analysis was performed on significatively up- and downregulated genes using the PANTHER Classification System's over-representation test tool (version 17.0) (GO Ontology database, released 2022-03-22, doi:10.5281/zenodo.6399963; [8]). Fischer's Exact Test with FDR correction was used and significant GO were determined with *p* < 0.05.

For RNA sequencing, near confluent HEK-293T and HeLa cells were suspended in DMEM low glucose supplemented as above because MiR05 is unsuitable for long-term culture. Cells were treated with 10 μM MTALTND4 or water for 4 or 72 h as in Ref. [3], with each condition replicated three times (n = 3 for each condition). Total RNA was then extracted from both control and treated cells, as above. RNA quantity and purity were also assessed as above. RNA samples were sent to the “Institut de Recherche en Immunologie et en Cancérologie- IRIC” (Montréal, QC, CA) for sequencing. Raw 84 bp paired-end reads were imported into the Galaxy (version 23.0) web-based platform [9]: (i) quality control was performed using FastQC; (ii) adaptors were trimmed and small reads (<15 nucleotides) were discarded using fastp with default settings; (iii) trimmed reads were mapped to the human reference genome GRCh38.p13 containing basic gene annotation, using RNA-Star with default settings; (iv) StringTie and StringTie merge were employed with the forward strand option to improve mapped read accuracy; (v) gene expression was quantified using featureCounts at the gene level (i.e. without distinguishing between isoforms) with the forward strand option and StringTie merge files as gene annotation; (vi) differential gene expression analysis was conducted using limma (logCPM normalization, Log2FC ≥ 1.0, adjusted p < 0.05). Gene Ontology analysis was conducted as above. Gene IDs were compared to the PANTHER *Homo sapiens* reference genome. Fischer's Exact Test, with FDR correction, was used for statistical analyses.

For RT-qPCR, cells were treated with 10 μM MTALTND4 or water for 4 h in MiR05 or DMEM low glucose. A total of six RNA samples were obtained for each culture medium (3 treatments and 3 controls). RT-qPCR analysis of selected genes was conducted at IRIC. RNA integrity was assessed using a Bioanalyzer 2100 (Agilent, Santa Clara, CA, USA). Samples were reverse transcribed using the Maxima First Strand cDNA synthesis kit with ds DNase (Thermo Fisher Scientific, San Jose, CA, USA). Gene expression was determined using assays designed with the Universal Probe Library from Roche (www.universalprobelibrary.com). For each assay, a standard curve was generated to confirm that the assay efficiency was between 90 % and 110 %. The QuantStudio qPCR instrument (Thermo Fisher Scientific) was used to detect the amplification level. “Control” and “treated” Ct values were normalized and compared using the 2^ΔCt^ method [10]. GAPDH and ACTB were selected as housekeeping. Statistical significance (p < 0.05) was determined via unpaired two-tailed Student's t-test in GraphPad Prism (GraphPad Software Inc., San Diego, CA, USA).

## 3 Results and discussion

**Fig. 1:**
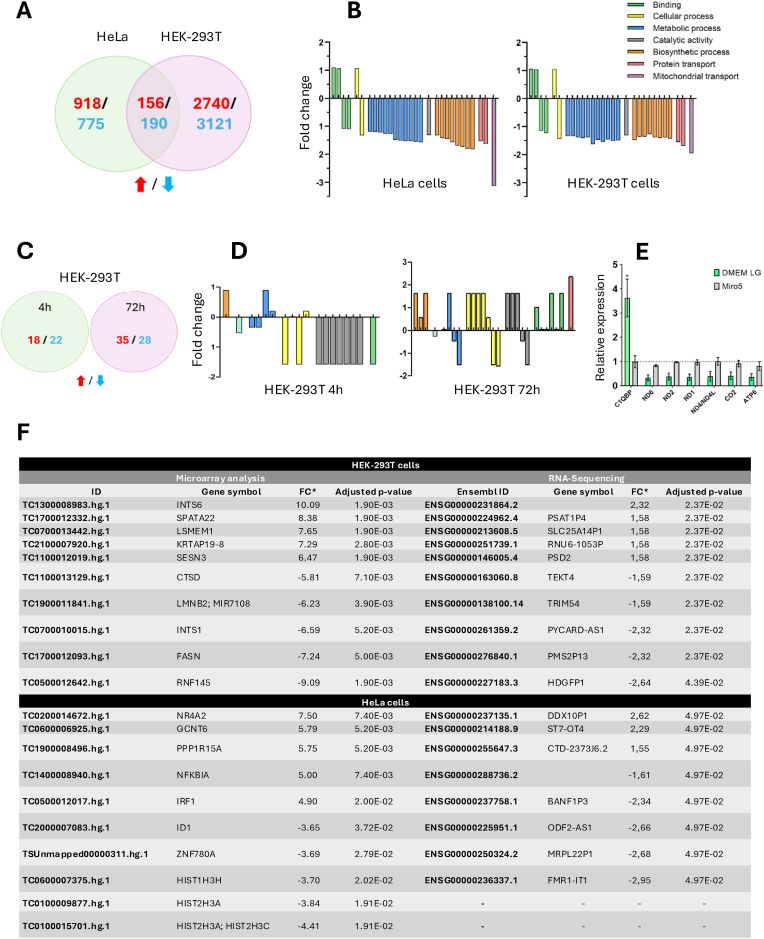
**Effect of MTALTND4 on gene expression in HeLa and HEK-293T cells.** Microarray analysis (A and B) was performed on HeLa (n = 2) and HEK-293T cells (n = 2) treated with MTALTND4 (10 μM) for 4 h in MiR05. RNA sequencing (C and D) was performed on HEK-293T cells treated exogenously with MTALTND4 (10 μM) for 4 (n = 3) and 72 h (n = 3) in DMEM low glucose. (A) Venn diagram depicting upregulated (red) and downregulated (blue) genes in both cell types after microarray analysis (adjusted p-value <0.05); (B) Common Gene Ontology results of differentially expressed genes in both cell types after microarray analysis; (C) Upregulated (red) and downregulated (blue) genes after 4- and 72-h exposure in HEK-293T cells after RNA sequencing (adjusted p-value <0.05); (D) Gene ontology analysis of differentially expressed coding genes in HEK-293T cells after RNA sequencing. No significant GO were found after Fischer's Exact Test, with FDR correction; (E) HEK-293T cells were subjected to the MTALTND4 peptide for a 4-h exposure in two distinct culture media, DMEM low glucose and MiR05. Relative expression values of treated cells can be compared against the average relative expression of the untreated condition, which serves as control and is normalized to 1, depicted by a dotted line. Data shown as mean ± SD of three biological replicates (n = 3). Student's t-test, ∗p < 0.05; (F) Five most upregulated and downregulated genes identified with microarray analysis and RNA sequencing after a 4-h exposure to exogenous MATLND4 (10 μM) in HeLa and HEK-293T cells.

Our initial goal was to determine whether MTALTND4, which we had previously shown to affect cellular physiology [5], also alters nuclear gene expression in a manner comparable to other MDPs such as Humanin and MOTS-c [1]. Using microarrays in MiR05 medium, a high number of differentially expressed genes (DEGs) was detected in two cell lines (HeLa: 918 upregulated and 775 downregulated; HEK-293T: 2740 upregulated and 3121 downregulated), many of which were linked to metabolism, biosynthesis, and transport (Fig. 1A, Appendix A Tables S1–3). The findings were consistent with the bioenergetic depression previously observed in MTALTND4-treated cells [5]. The effect was more pronounced in HEK-293T cells, which were also previously shown to be more sensitive to MTALTND4 [5].

However, RNA-seq analysis, conducted in DMEM low glucose and with longer peptide exposure, detected very few DEGs (less than 35, with no overlap between the 4-h and 72-h data or between the two cell lines) and almost no overlap with the microarray results (only two DEGs, PYCARD-AS1 and DRC1, were common between the microarray and RNA-seq datasets). The small magnitude of fold changes (<2 for RNA-seq) and the absence of reproducibility across methods suggest that MTALTND4's impact on the transcriptome is context-dependent and may be indistinguishable from background noise in certain experimental settings. These discrepancies may be due to inherent technical differences. Microarray analyses are highly dependent on probe selection and binding specificity, with background signals and cross-hybridization potentially leading to significant biases when measuring differential gene expression (e.g. Ref. [11]). In contrast, RNA-seq is generally considered more sensitive, particularly for genes with low expression levels, though factors such as sample preparation and computational analysis can substantially influence the results [11].

A major biological variable in this study was the use of different culture media: MiR05 for microarrays and DMEM low glucose for RNA-seq. MiR05 supports mitochondrial function during short-term assays but is not optimized for cell proliferation [7], whereas DMEM supports cellular growth but may differently regulate mitochondrial activity. These differences likely contribute to the divergent transcriptomic signatures observed. For example, C1QBP, a known MTALTND4 interactor and a regulator of mitochondrial genes and metabolism [5], was upregulated in DMEM low glucose as measured by RT-qPCR, but not in MiR05 (Fig. 1E–F). While this upregulation in DMEM aligns with our previous work suggesting a role for MTALTND4 in mitochondrial function [5], the RT-qPCR data indicate that MTALTND4's effects are influenced by the medium's composition. This phenomenon, which has been documented previously [[12], [13], [14]], may alter gene expression pathways, complicating direct comparisons across methods. These findings underline the need for standardization of culture conditions in future studies to minimize confounding effects that could obscure the true impact of MTALTND4 on gene expression. Additionally, RT-qPCR validation of three top candidate genes from the microarray analysis (LMNB2, FASN, and NFKBIA) revealed no significant expression changes in either MiR05 or DMEM low glucose (Fig. 1E–F), further emphasizing the inconsistency between methods. Given that RT-qPCR is often considered the gold standard for validating gene expression changes, the lack of reproducibility with the microarray data raises concerns about the reliability of the microarray findings. Microarrays may be more susceptible to overestimating fold changes, especially in the presence of non-specific hybridization.

In summary, given the current limitations, our results should be interpreted with caution. While microarrays identified many DEGs consistent with our prior physiological study showing MTALTND4 induces bioenergetic depression [5], these findings were not consistently validated by RNA-seq or RT-qPCR, suggesting that the microarray results may be influenced by technical biases and culture-specific effects. This is consistent with the observation that reproducibility across methods like microarrays, RNA-seq, and RT-qPCR can be limited, particularly for genes with low fold changes (<2) [15], as observed in the present study. Our results caution against overinterpreting transcriptomic changes from a single method or culture condition and underscore the need for standardized conditions and multi-platform validation. MTALTND4's potential bioactivity remains to be fully elucidated, particularly in relation to its interaction with C1QBP, a key regulator of mitochondrial function. Future studies should aim to standardize experimental conditions, explore alternative culture media, and incorporate more rigorous validation approaches, such as functional assays, to better understand the underlying mechanisms of MTALTND4's effects on gene expression.

## CRediT authorship contribution statement

**Ludovic Nadeau-Lachance:** Writing – original draft, Visualization, Validation, Investigation, Formal analysis. **Thierry Choquette:** Writing – review & editing, Visualization, Validation, Investigation, Formal analysis. **Hajar Hosseini Khorami:** Resources, Methodology, Formal analysis. **Annie Angers:** Writing – review & editing, Resources, Methodology, Formal analysis. **Sophie Breton:** Writing – review & editing, Writing – original draft, Visualization, Validation, Supervision, Resources, Methodology, Funding acquisition, Formal analysis, Conceptualization.

## Funding

We acknowledge the funding by the Natural Sciences and Engineering Research Council of Canada grant RGPIN-2019–04076 (to SB) and Canada Research Chair in Mitochondrial Evolutionary Biology (Tier 2) (to SB).

## Declaration of competing interest

The authors declare that they have no known competing financial interests or personal relationships that could have appeared to influence the work reported in this paper.

## Data Availability

Data will be made available on request.

## Appendix A Supplementary data

The Supporting Information includes tables of differentially expressed genes in HeLa and HEK-293T cells after microarrays and RNA sequencing analyses.
