# Supplementary material for: Evaluating the effect of the mitochondrial alternative peptide MTALTND4 on gene expression
Source: Biochem Biophys Rep. 2025 Aug 27;44:102223. doi: 10.1016/j.bbrep.2025.102223 (PMC12409802; doi:10.1016/j.bbrep.2025.102223)
Supplement: Multimedia component 1 [file mmc1.docx]

**Appendix A**

**Table S1**. Upregulated and downregulated genes between control and treated HeLa cells cultured in MiR05 medium and identified from microarray analysis.

🡪 See link below

**Table S2**. Upregulated and downregulated genes between control and treated HEK-293T cells cultured in MiR05 medium and identified from microarray analysis.

🡪 See link below

Zenodo link:

https://zenodo.org/records/13376073?token=eyJhbGciOiJIUzUxMiJ9.eyJpZCI6IjhiNTM0MTgzLWIzN2EtNGQxOS1hMWExLWFlNGZiZjIwYTljMCIsImRhdGEiOnt9LCJyYW5kb20iOiIyMDdlZjA3Yzk3OTQ0ZmY0MzRmZjQ2MTIyNjg0MmQyYSJ9.ZifxJf7x9k36f_kKRmpl4FLCVf6IGx7WTA9_exTlUPaoPrqLiUOjGF7qRJjCyZ5B5-24tVxitUzi1_UkjZWWbw

**Table S3**. Common **(A)** upregulated and **(B)** downregulated genes between HeLa and HEK-293T cells cultured in MiR05 medium and identified from microarray analysis.

| **A. COMMON UPREGULATED GENES** | | | | | | |
| --- | --- | --- | --- | --- | --- | --- |
| **ID** | **Description** | **Gene Symbol** | **HeLa** | | **HEK** | |
|  |  |  | **Fold change** | **P-value** | **Fold change** | **P-value** |
| TC1800008235.hg.1 | Transcript Identified by aceview, Entrez Gene ID(s) 171586 | ABHD3 | 1.88 | 9.10E-03 | 1.8 | 1.32E-02 |
| TC0500011296.hg.1 | Acyl-coa thioesterase 12 | ACOT12 | 1.9 | 1.86E-02 | 2.47 | 2.70E-03 |
| TC2000008950.hg.1 | Memczak2013 ANTISENSE, CDS, coding, INTERNAL best transcript NM_001076552 | ACSS2 | 1.92 | 1.25E-02 | 1.74 | 2.74E-02 |
| TC2000007202.hg.1 | Acyl-coa synthetase short-chain family member 2 | ACSS2 | 1.79 | 9.20E-03 | 1.64 | 2.15E-02 |
| TC1100006831.hg.1 | Adrenomedullin | ADM | 2.53 | 2.00E-04 | 1.91 | 3.20E-03 |
| TC1100011083.hg.1 | AHNAK nucleoprotein | AHNAK | 1.56 | 4.21E-02 | 1.79 | 1.15E-02 |
| TC0200015424.hg.1 | Amyotrophic lateral sclerosis 2 chromosome region candidate 12 | ALS2CR12 | 1.99 | 1.60E-03 | 1.55 | 2.19E-02 |
| TC0100015350.hg.1 | Adenosine monophosphate deaminase 1 | AMPD1 | 1.93 | 2.40E-03 | 4.44 | 2.83E-06 |
| TC1300008084.hg.1 | Atpase, class VI, type 11A | ATP11A | 1.84 | 1.80E-03 | 1.79 | 3.80E-02 |
| TC1400010759.hg.1 | Atpase, H+ transporting, lysosomal 34kda, V1 subunit D | ATP6V1D | 1.81 | 1.58E-02 | 2.36 | 1.80E-03 |
| TC0300013025.hg.1 | Butyrylcholinesterase | BCHE | 2 | 3.00E-03 | 2.11 | 1.80E-03 |
| TC2000010009.hg.1 | Bladder cancer associated protein | BLCAP | 1.56 | 3.14E-02 | 2.08 | 2.00E-03 |
| TC1500007304.hg.1 | Chromosome 15 open reading frame 65 | C15orf65 | 1.65 | 2.52E-02 | 3.31 | 7.37E-05 |
| TC0500013298.hg.1 | C1q and tumor necrosis factor related protein 3 | C1QTNF3 | 2.25 | 1.50E-03 | 3.84 | 2.53E-05 |
| TC0600013144.hg.1 | Transcript Identified by aceview, Entrez Gene ID(s) 93663; 253582; Jeck2013 ALT_ACCEPTOR, ALT_DONOR, downstream_end, ncrna, OVEXON, upstream_start best transcript TCONS_l2_00025470; Jeck2013 ALT_ACCEPTOR, ncrna, OVEXON, upstream_start best transcript TCONS_l2_00024911; Salzman2013 ANTISENSE, CDS, coding, downstream_end, intronic, OVCODE, OVEXON, UTR3 best transcript NM_000426; Salzman2013 ANNOTATED, INTERNAL, ncrna, OVEXON best transcript TCONS_l2_00025470; putative novel transcript; novel transcript | C6orf191andARHGAP18; RP1-69D17.4; RP1-69D17.3; TCONS_l2_00025470; TCONS_l2_00024911; LAMA2 | 1.55 | 2.38E-02 | 2.35 | 4.00E-04 |
| TC1600011351.hg.1 | Jeck2013 ANTISENSE, CDS, coding, INTERNAL, OVCODE, OVEXON best transcript NM_001042476 | CARHSP1 | 1.94 | 2.90E-03 | 1.51 | 3.69E-02 |
| TC1400010776.hg.1 | Catsper channel auxiliary subunit beta | CATSPERB | 1.74 | 3.80E-03 | 2.93 | 2.20E-05 |
| TC1100010472.hg.1 | Coiled-coil domain containing 73 | CCDC73 | 2.83 | 2.00E-04 | 1.52 | 4.89E-02 |
| TC1100008665.hg.1 | Coiled-coil domain containing 81 | CCDC81 | 2.68 | 9.36E-05 | 2.81 | 6.13E-05 |
| TC0400012818.hg.1 | Cyclin G2 | CCNG2 | 1.68 | 1.30E-02 | 2.51 | 3.00E-04 |
| TC0400011043.hg.1 | Cyclin-dependent kinase-like 2 (CDC2-related kinase) | CDKL2 | 2.77 | 6.84E-05 | 1.74 | 6.10E-03 |
| TC1700012361.hg.1 | CMT1A duplicated region transcript 1 | CDRT1 | 1.78 | 2.00E-02 | 2.66 | 8.00E-04 |
| TC1600007985.hg.1 | Cholesteryl ester transfer protein, plasma | CETP | 2.19 | 7.00E-04 | 3.45 | 1.64E-05 |
| TC0600014153.hg.1 | Cilia and flagella associated protein 206 | CFAP206 | 2.24 | 1.20E-03 | 1.83 | 7.50E-03 |
| TC1200008686.hg.1 | Transcript Identified by aceview, Entrez Gene ID(s) 50515 | CHST11 | 1.68 | 3.84E-02 | 1.58 | 2.06E-02 |
| TC0200015397.hg.1 | CDC like kinase 1 | CLK1 | 1.79 | 1.92E-02 | 2.49 | 1.40E-03 |
| TC0500013020.hg.1 | CDC like kinase 4 | CLK4 | 1.92 | 2.80E-03 | 1.51 | 3.42E-02 |
| TC1400006828.hg.1 | Cochlin | COCH | 1.75 | 4.96E-02 | 1.9 | 2.81E-02 |
| TC1600008889.hg.1 | Copine VII | CPNE7 | 1.83 | 3.95E-02 | 2.89 | 1.90E-03 |
| TC0300010770.hg.1 | Cysteine-serine-rich nuclear protein 1 | CSRNP1 | 3.44 | 2.58E-05 | 1.63 | 1.81E-02 |
| TC0800008481.hg.1 | Collagen triple helix repeat containing 1 | CTHRC1 | 1.76 | 2.32E-02 | 2.5 | 1.30E-03 |
| TC0200014414.hg.1 | Chemokine (C-X-C motif) receptor 4 | CXCR4 | 2.42 | 3.00E-04 | 1.96 | 2.30E-03 |
| TC1000012480.hg.1 | Cytochrome P450, family 2, subfamily C, polypeptide 18 | CYP2C18 | 1.65 | 2.09E-02 | 3.9 | 1.63E-05 |
| TC1000011478.hg.1 | Cytochrome P450, family 2, subfamily C, polypeptide 8 | CYP2C8 | 1.65 | 3.27E-02 | 1.9 | 9.70E-03 |
| TC0400007495.hg.1 | DCN1, defective in cullin neddylation 1, domain containing 4 | DCUN1D4 | 1.57 | 4.16E-02 | 3.57 | 4.89E-05 |
| TC1200010968.hg.1 | DNA-damage-inducible transcript 3 | DDIT3 | 1.78 | 1.53E-02 | 2.89 | 3.00E-04 |
| TC0800009094.hg.1 | DENN/MADD domain containing 3 | DENND3 | 2.29 | 6.00E-04 | 1.8 | 9.50E-03 |
| TSUnmapped00000088.hg.1 | Dual specificity phosphatase 16 | DUSP16 | 1.54 | 4.25E-02 | 2.11 | 2.20E-03 |
| TC1200009967.hg.1 | Dual specificity phosphatase 16 | DUSP16 | 1.63 | 2.44E-02 | 1.87 | 6.60E-03 |
| TC1900008456.hg.1 | Epididymal sperm binding protein 1 | ELSPBP1 | 1.83 | 2.90E-03 | 2.34 | 3.00E-04 |
| TC0800011621.hg.1 | Ectonucleotide pyrophosphatase/phosphodiesterase 2 | ENPP2 | 1.56 | 4.88E-02 | 1.56 | 4.80E-02 |
| TC2100008143.hg.1 | V-ets avian erythroblastosis virus E26 oncogene homolog | ERG | 1.82 | 3.62E-02 | 1.91 | 2.57E-02 |
| TC0100018403.hg.1 | ERBB receptor feedback inhibitor 1 | ERRFI1 | 1.65 | 1.36E-02 | 1.61 | 1.72E-02 |
| TC0700011318.hg.1 | Endogenous retrovirus group 3, member 1; zinc finger protein 117 | ERV3-1; ZNF117 | 1.67 | 1.35E-02 | 1.73 | 9.60E-03 |
| TC2000009945.hg.1 | Family with sequence similarity 209, member A | FAM209A | 1.87 | 9.50E-03 | 2.12 | 3.20E-03 |
| TC0700009060.hg.1 | Family with sequence similarity 71, member F1 | FAM71F1 | 1.63 | 4.05E-02 | 2.36 | 1.80E-03 |
| TC0300008532.hg.1 | F-box protein 40 | FBXO40 | 1.63 | 9.80E-03 | 1.81 | 3.10E-03 |
| TC1500010853.hg.1 | Fibrous sheath interacting protein 1 | FSIP1 | 1.69 | 4.69E-02 | 1.78 | 3.11E-02 |
| TC1900006588.hg.1 | Growth arrest and DNA-damage-inducible, beta | GADD45B | 2.19 | 8.00E-04 | 2.06 | 1.40E-03 |
| TC0600012064.hg.1 | Glial cells missing homolog 1 (Drosophila) | GCM1 | 1.93 | 7.60E-03 | 2.16 | 2.80E-03 |
| TC0600012502.hg.1 | Gap junction protein beta 7 | GJB7 | 1.54 | 1.60E-02 | 1.52 | 1.95E-02 |
| TC0500007465.hg.1 | GC-rich promoter binding protein 1 | GPBP1 | 1.69 | 9.40E-03 | 3.26 | 2.30E-05 |
| TC1100012000.hg.1 | G protein-coupled receptor 83 | GPR83 | 2.05 | 1.90E-03 | 2.78 | 1.00E-04 |
| TC1200009980.hg.1 | G protein-coupled receptor, class C, group 5, member D | GPRC5D | 2.85 | 1.00E-04 | 2.39 | 4.00E-04 |
| TC0600013341.hg.1 | GVQW motif containing 2 | GVQW2 | 1.55 | 2.78E-02 | 1.64 | 1.57E-02 |
| TC0100009608.hg.1 | Hydroxyacid oxidase 2 (long chain) | HAO2 | 1.72 | 3.94E-02 | 2.64 | 1.60E-03 |
| TC0700008747.hg.1 | HMG-box transcription factor 1 | HBP1 | 1.75 | 3.80E-03 | 2.52 | 9.22E-05 |
| TC1200012248.hg.1 | Hydroxycarboxylic acid receptor 3 | HCAR3 | 2.03 | 7.50E-03 | 1.77 | 2.23E-02 |
| TC0300012166.hg.1 | Hematopoietic cell-specific Lyn substrate 1 | HCLS1 | 1.75 | 2.47E-02 | 1.65 | 3.96E-02 |
| TC1700008033.hg.1 | Hexamethylene bis-acetamide inducible 1 | HEXIM1 | 2.01 | 1.30E-03 | 2.67 | 9.18E-05 |
| TC0600011125.hg.1 | Histone cluster 1, h2ab | HIST1H2AB | 1.76 | 3.40E-03 | 4.92 | 5.81E-07 |
| TC0600011133.hg.1 | Memczak2013 ANTISENSE, CDS, coding, upstream_start, UTR3, UTR5 best transcript NM_003523 | HIST1H2BE | 3.95 | 1.30E-05 | 1.87 | 5.50E-03 |
| TC0100015698.hg.1 | Histone cluster 2, h3d | HIST2H3D | 1.6 | 2.48E-02 | 2.03 | 2.60E-03 |
| TSUnmapped00000141.hg.1 | Hydroxymethylbilane synthase | HMBS | 2.28 | 1.10E-03 | 2.08 | 2.50E-03 |
| TC0100008503.hg.1 | Hook microtubule-tethering protein 1 | HOOK1 | 2.15 | 1.03E-02 | 2.31 | 6.20E-03 |
| TC2200007043.hg.1 | HORMA domain containing 2 | HORMAD2 | 1.72 | 1.73E-02 | 2.59 | 5.00E-04 |
| TC1900006977.hg.1 | Intercellular adhesion molecule 1 | ICAM1 | 2.33 | 2.00E-03 | 2.13 | 4.20E-03 |
| TC0100008653.hg.1 | Interleukin 12 receptor, beta 2 | IL12RB2 | 1.6 | 1.95E-02 | 3.56 | 1.52E-05 |
| TSUnmapped00000135.hg.1 | Inositol polyphosphate-5-phosphatase D | INPP5D | 1.75 | 7.00E-03 | 1.77 | 2.20E-03 |
| TC2100006784.hg.1 | Junctional adhesion molecule 2 | JAM2 | 1.63 | 3.04E-02 | 1.68 | 2.33E-02 |
| TSUnmapped00000492.hg.1 | K(lysine) acetyltransferase 6B | KAT6B | 1.68 | 5.30E-03 | 1.61 | 9.00E-03 |
| TSUnmapped00000282.hg.1 | K(lysine) acetyltransferase 6B | KAT6B | 1.66 | 1.64E-02 | -1.65 | 1.70E-02 |
| TC0300009673.hg.1 | Kelch-like family member 24 | KLHL24 | 1.8 | 6.10E-03 | 1.98 | 2.40E-03 |
| TC1600011368.hg.1 | Linker for activation of T-cells | LAT | 1.56 | 4.35E-02 | 1.58 | 4.05E-02 |
| TC1100007005.hg.1 | Lactate dehydrogenase C | LDHC | 1.84 | 1.52E-02 | 2.4 | 1.80E-03 |
| TC1400010748.hg.1 | Long intergenic non-protein coding RNA 1588 | LINC01588 | 1.68 | 4.04E-02 | 2.51 | 1.80E-03 |
| TC1200012778.hg.1 | Lamin tail domain containing 1 | LMNTD1 | 1.65 | 2.09E-02 | 1.63 | 2.45E-02 |
| TC1200009198.hg.1 | Leucine rich repeat containing 43 | LRRC43 | 2.2 | 1.40E-03 | 2.53 | 4.00E-04 |
| TC0700013442.hg.1 | Leucine-rich single-pass membrane protein 1 | LSMEM1 | 2.59 | 3.00E-04 | 7.65 | 3.28E-07 |
| TC0100014769.hg.1 | Mucolipin 3 | MCOLN3 | 1.98 | 1.10E-03 | 1.85 | 2.30E-03 |
| TC1700012310.hg.1 | Major facilitator superfamily domain containing 11 | MFSD11 | 1.79 | 2.22E-02 | 1.69 | 3.56E-02 |
| TC1700008598.hg.1 | Mast cell immunoglobulin-like receptor 1 | MILR1 | 1.68 | 2.81E-02 | 1.81 | 1.48E-02 |
| TC0200016770.hg.1 | Microrna 6809; tensin 1 | MIR6809; TNS1 | 1.87 | 4.75E-02 | 2.11 | 2.21E-02 |
| TC1000006912.hg.1 | Mannose receptor, C type 1 | MRC1 | 2.01 | 3.50E-03 | 1.89 | 6.40E-03 |
| TC1100010998.hg.1 | Membrane-spanning 4-domains, subfamily A, member 4E | MS4A4E | 1.61 | 3.06E-02 | 2.07 | 3.10E-03 |
| TC0200012977.hg.1 | Memczak2013 ANTISENSE, coding, INTERNAL, intronic best transcript NM_001202514 | MXD1 | 3.43 | 1.45E-05 | 2.19 | 6.00E-04 |
| TC0200009927.hg.1 | Myosin IIIB | MYO3B | 1.64 | 4.90E-02 | 2.7 | 1.00E-03 |
| TC0200009927.hg.1 | Myosin IIIB | MYO3B | 1.64 | 4.90E-02 | 2.7 | 1.00E-03 |
| TC1400008940.hg.1 | Nuclear factor of kappa light polypeptide gene enhancer in B-cells inhibitor, alpha | NFKBIA | 5 | 2.06E-06 | 3.06 | 5.70E-05 |
| TC0300013855.hg.1 | Nuclear factor of kappa light polypeptide gene enhancer in B-cells inhibitor, zeta | NFKBIZ | 2.26 | 7.00E-04 | 1.74 | 8.80E-03 |
| TC0200014672.hg.1 | Nuclear receptor subfamily 4, group A, member 2 | NR4A2 | 7.5 | 1.83E-06 | 1.86 | 1.54E-02 |
| TC0900008219.hg.1 | Nuclear receptor subfamily 4, group A, member 3 | NR4A3 | 2.22 | 4.00E-04 | 1.94 | 1.40E-03 |
| TC0900011199.hg.1 | Olfactory receptor, family 2, subfamily K, member 2 | OR2K2 | 1.79 | 1.23E-02 | 2.93 | 2.00E-04 |
| TC1100007637.hg.1 | Olfactory receptor, family 8, subfamily U, member 1; olfactory receptor, family 8, subfamily U, member 8 | OR8U1; OR8U8 | 1.72 | 1.13E-02 | 1.98 | 3.00E-03 |
| TC1100009833.hg.1 | Memczak2013 ALT_ACCEPTOR, ALT_DONOR, coding, INTERNAL, intronic best transcript NM_020896 | OSBPL5 | 1.69 | 7.50E-03 | 1.97 | 1.40E-03 |
| TC1100011310.hg.1 | Pyruvate carboxylase | PC | 2.07 | 1.30E-03 | 1.75 | 3.14E-02 |
| TC1600007007.hg.1 | Pyridoxal-dependent decarboxylase domain containing 1 | PDXDC1 | 1.55 | 4.25E-02 | 1.89 | 6.40E-03 |
| TC0500007641.hg.1 | Phosphoinositide-3-kinase, regulatory subunit 1 (alpha) | PIK3R1 | 1.99 | 2.10E-02 | 2.27 | 8.60E-03 |
| TC0100014040.hg.1 | Phosphoinositide-3-kinase, regulatory subunit 3 (gamma) | PIK3R3 | 1.65 | 1.52E-02 | 2.67 | 2.00E-04 |
| TC0100018553.hg.1 | Pleckstrin homology domain containing, family A member 6 | PLEKHA6 | 2.18 | 3.50E-03 | 1.76 | 4.73E-02 |
| TC0600008757.hg.1 | Proline-rich nuclear receptor coactivator 1 | PNRC1 | 2.15 | 2.00E-03 | 1.79 | 1.07E-02 |
| TC1200012534.hg.1 | Transcript Identified by aceview, Entrez Gene ID(s) 5426 | POLE | 1.77 | 1.95E-02 | 1.76 | 2.04E-02 |
| TC0600011372.hg.1 | Protein phosphatase 1, regulatory subunit 10 | PPP1R10 | 3.56 | 1.96E-05 | 1.62 | 1.86E-02 |
| TC0200010165.hg.1 | Protein phosphatase 1, regulatory (inhibitor) subunit 1C | PPP1R1C | 1.62 | 1.17E-02 | 2.03 | 1.10E-03 |
| TC1400007444.hg.1 | Protein phosphatase 1, regulatory subunit 36 | PPP1R36 | 1.59 | 3.51E-02 | 2.68 | 4.00E-04 |
| TC1100011182.hg.1 | Phosphorylase, glycogen, muscle | PYGM | 1.81 | 5.50E-03 | 2.13 | 1.10E-03 |
| TC0200015476.hg.1 | Ras association (ralgds/AF-6) and pleckstrin homology domains 1 | RAPH1 | 1.94 | 1.70E-03 | 2.07 | 9.00E-04 |
| TC0700012099.hg.1 | Salzman2013 ALT_ACCEPTOR, ALT_DONOR, coding, INTERNAL, intronic best transcript NM_006989 | RASA4 | 1.7 | 1.08E-02 | 1.66 | 1.39E-02 |
| TC0100013636.hg.1 | Ring finger protein 19B | RNF19B | 1.6 | 1.91E-02 | 2.36 | 4.00E-04 |
| TSUnmapped00000289.hg.1 | Ribosomal protein l7a | RPL7A | 1.57 | 1.27E-02 | 1.58 | 1.17E-02 |
| TC0700010181.hg.1 | Radial spoke head 10 homolog B2 (Chlamydomonas); radial spoke head 10 homolog B (Chlamydomonas) | RSPH10B2; RSPH10B | 1.64 | 3.38E-02 | 2.06 | 4.80E-03 |
| TC0200016673.hg.1 | Reticulon 4 | RTN4 | 1.52 | 2.32E-02 | 1.73 | 5.80E-03 |
| TC1800007201.hg.1 | Sialic acid binding Ig-like lectin 15 | SIGLEC15 | 1.68 | 9.40E-03 | 1.73 | 7.00E-03 |
| TSUnmapped00000474.hg.1 | Solute carrier family 16 (monocarboxylate transporter), member 1 | SLC16A1 | 1.62 | 2.28E-02 | 2.61 | 3.00E-04 |
| TC0900010910.hg.1 | Solute carrier family 35 (UDP-glcnac/UDP-glucose transporter), member D2 | SLC35D2 | 1.93 | 3.70E-03 | 6.14 | 7.60E-07 |
| TC1200009547.hg.1 | Solute carrier family 6 (neurotransmitter transporter), member 13 | SLC6A13 | 1.56 | 4.15E-02 | 3.28 | 7.63E-05 |
| TC2000007670.hg.1 | Snail family zinc finger 1 | SNAI1 | 1.58 | 2.76E-02 | 1.52 | 3.87E-02 |
| TC0400008624.hg.1 | Transcript Identified by aceview, Entrez Gene ID(s) 166378 | SPATA5 | 1.87 | 7.80E-03 | 1.69 | 9.20E-03 |
| TC0200016423.hg.1 | Speedy/RINGO cell cycle regulator family member A | SPDYA | 1.53 | 2.15E-02 | 3.4 | 1.07E-05 |
| TC0100010905.hg.1 | SWT1 RNA endoribonuclease homolog | SWT1 | 1.83 | 5.30E-03 | 2.18 | 1.00E-03 |
| TC0100015445.hg.1 | T-box 15 | TBX15 | 1.63 | 3.70E-02 | 2.08 | 4.50E-03 |
| TC0100011621.hg.1 | Transforming growth factor beta 2; TGFB2 overlapping transcript 1 | TGFB2; TGFB2-OT1 | 1.69 | 6.00E-03 | 1.59 | 1.18E-02 |
| TSUnmapped00000172.hg.1 | Transglutaminase 4 | TGM4 | 1.56 | 3.40E-02 | 1.72 | 2.17E-02 |
| TC0900010388.hg.1 | Transcript Identified by aceview, Entrez Gene ID(s) 23670 | TMEM2 | 2.48 | 1.00E-03 | 2.95 | 1.87E-05 |
| TC1000010744.hg.1 | Transmembrane protein 26 | TMEM26 | 1.52 | 2.63E-02 | 1.5 | 2.98E-02 |
| TC0400010895.hg.1 | Transmembrane protease, serine 11D | TMPRSS11D | 1.83 | 1.30E-02 | 2.76 | 4.00E-04 |
| TC0600009597.hg.1 | Tumor necrosis factor, alpha-induced protein 3 | TNFAIP3 | 4.71 | 5.73E-07 | 1.62 | 7.50E-03 |
| TC1800007508.hg.1 | Transcript Identified by aceview, Entrez Gene ID(s) 8792 | TNFRSF11A | 2.03 | 1.40E-03 | 2.03 | 1.40E-03 |
| TC1900006804.hg.1 | Tumor necrosis factor (ligand) superfamily, member 9 | TNFSF9 | 1.86 | 5.70E-03 | 1.93 | 3.90E-03 |
| TC1100013150.hg.1 | Tryptophan hydroxylase 1 | TPH1 | 1.65 | 2.03E-02 | 2.05 | 2.60E-03 |
| TC1700012333.hg.1 | Transient receptor potential cation channel, subfamily V, member 3 | TRPV3 | 1.84 | 1.70E-03 | 1.56 | 1.15E-02 |
| TC0200013602.hg.1 | Testis specific 10 | TSGA10 | 1.84 | 6.00E-03 | 1.59 | 2.44E-02 |
| TC1800006655.hg.1 | Thioredoxin domain containing 2 (spermatozoa) | TXNDC2 | 2.43 | 2.10E-03 | 2.05 | 7.80E-03 |
| TC1900009984.hg.1 | Unc-13 homolog A (C. Elegans) | UNC13A | 1.62 | 3.34E-02 | 1.62 | 3.34E-02 |
| TC0200007722.hg.1 | Memczak2013 ANTISENSE, CDS, coding, INTERNAL best transcript NM_014709 | USP34 | 2.18 | 2.50E-03 | 2.29 | 5.00E-04 |
| TC1100008480.hg.1 | UV radiation resistance associated | UVRAG | 1.75 | 6.20E-03 | 1.92 | 4.19E-02 |
| TC1900012055.hg.1 | Vomeronasal 1 receptor 1 | VN1R1 | 1.75 | 8.00E-03 | 1.75 | 7.90E-03 |
| TC0200016676.hg.1 | WD repeat containing planar cell polarity effector | WDPCP | 1.52 | 4.21E-02 | 1.99 | 3.00E-03 |
| TC1000006466.hg.1 | WD repeat domain 37 | WDR37 | 1.54 | 3.79E-02 | 1.74 | 1.20E-02 |
| TC0300014077.hg.1 | WD repeat domain 49 | WDR49 | 1.8 | 4.50E-03 | 1.76 | 5.90E-03 |
| TC1200012719.hg.1 | WD repeat domain 66 | WDR66 | 1.73 | 4.74E-02 | 2.38 | 4.70E-03 |
| TC0200007132.hg.1 | Yippee like 5 | YPEL5 | 2.04 | 4.40E-03 | 2.91 | 3.00E-04 |
| TC0500013282.hg.1 | Zinc finger, DHHC-type containing 11 | ZDHHC11 | 1.83 | 3.27E-02 | 1.78 | 3.61E-02 |
| TC1900012020.hg.1 | Zinc finger protein 432 | ZNF432 | 1.71 | 1.65E-02 | 1.58 | 3.41E-02 |
| TC0500009673.hg.1 | Zinc finger protein 454 | ZNF454 | 2.16 | 1.20E-03 | 1.98 | 2.90E-03 |
| TC1900011920.hg.1 | Zinc finger protein 98 | ZNF98 | 1.55 | 3.04E-02 | 1.53 | 3.33E-02 |

| **B. COMMON DOWNREGULATED GENES** | | | | | | |
| --- | --- | --- | --- | --- | --- | --- |
| **ID** | **Description** | **Gene Symbol** | **HeLa** | | **HEK** | |
|  |  |  | **Fold change** | **P-value** | **Fold change** | **P-value** |
| TC0700013065.hg.1 | ATP binding cassette subfamily F member 2 | ABCF2 | -1.52 | 4.80E-02 | -1.68 | 1.83E-02 |
| TC1400007647.hg.1 | Acyl-coa thioesterase 2 | ACOT2 | -1.57 | 4.44E-02 | -1.89 | 2.80E-03 |
| TC0200010577.hg.1 | ADAM metallopeptidase domain 23 | ADAM23 | -2.01 | 5.40E-03 | -1.98 | 6.00E-03 |
| TC1400010715.hg.1 | Ajuba LIM protein | AJUBA | -1.64 | 2.24E-02 | -1.51 | 4.87E-02 |
| TC1700007189.hg.1 | Aldehyde dehydrogenase 3 family, member A2 | ALDH3A2 | -1.5 | 3.18E-02 | -1.7 | 8.20E-03 |
| TC1100007384.hg.1 | Alkb homolog 3, alpha-ketoglutarate-dependent dioxygenase; SEC14-like 1 pseudogene 1 | ALKBH3; SEC14L1P1 | -1.61 | 2.96E-02 | -1.66 | 2.21E-02 |
| TC1000011050.hg.1 | Adaptor-related protein complex 3, mu 1 subunit | AP3M1 | -1.65 | 1.76E-02 | -1.73 | 1.11E-02 |
| TC0900009948.hg.1 | Rho guanine nucleotide exchange factor 39 | ARHGEF39 | -2.04 | 1.70E-03 | -3.03 | 5.25E-05 |
| TC0200016115.hg.1 | ADP-ribosylation factor like gtpase 4C | ARL4C | -1.69 | 1.65E-02 | -1.9 | 5.20E-03 |
| TC1200008213.hg.1 | Ataxin 7-like 3B | ATXN7L3B | -1.62 | 2.40E-02 | -3.23 | 5.88E-05 |
| TC1200011320.hg.1 | Bardet-Biedl syndrome 10 | BBS10 | -1.78 | 1.21E-02 | -2.2 | 1.80E-03 |
| TC0200010805.hg.1 | BCS1 homolog, ubiquinol-cytochrome c reductase complex chaperone | BCS1L | -1.78 | 7.50E-03 | -2.12 | 1.40E-03 |
| TC0X00010409.hg.1 | Brain expressed X-linked 2 | BEX2 | -1.68 | 2.47E-02 | -1.79 | 1.43E-02 |
| TC0700009996.hg.1 | Chromosome 7 open reading frame 50 | C7orf50 | -1.59 | 2.14E-02 | -1.55 | 2.81E-02 |
| TC0900010586.hg.1 | Chromosome 9 open reading frame 64 | C9orf64 | -2.67 | 2.50E-03 | -2.8 | 1.80E-03 |
| TC2100007032.hg.1 | Carbonyl reductase 1 | CBR1 | -1.69 | 1.01E-02 | -1.79 | 5.40E-03 |
| TC0300011054.hg.1 | Coiled-coil domain containing 71 | CCDC71 | -1.76 | 5.30E-03 | -3.83 | 6.13E-06 |
| TSUnmapped00000313.hg.1 | Coiled-coil domain containing 84 | CCDC84 | -2.1 | 5.40E-03 | -2.04 | 6.90E-03 |
| TSUnmapped00000085.hg.1 | Coiled-coil domain containing 84 | CCDC84 | -2.23 | 7.00E-04 | -1.5 | 3.68E-02 |
| TC1200006555.hg.1 | Cyclin D2 | CCND2 | -1.71 | 9.60E-03 | -1.99 | 2.10E-03 |
| TC0600011809.hg.1 | Cyclin D3 | CCND3 | -1.57 | 1.65E-02 | -1.93 | 1.70E-03 |
| TC0500012163.hg.1 | Cell division cycle 23 | CDC23 | -2.12 | 1.85E-02 | -1.92 | 3.59E-02 |
| TC0700010435.hg.1 | Cell division cycle associated 7-like | CDCA7L | -1.63 | 1.20E-02 | -1.63 | 1.20E-02 |
| TC0500012070.hg.1 | CDKN2A interacting protein N-terminal like | CDKN2AIPNL | -1.66 | 6.90E-03 | -1.71 | 4.90E-03 |
| TC0300013719.hg.1 | Centrosomal protein 19kda | CEP19 | -1.79 | 4.50E-03 | -1.67 | 9.60E-03 |
| TC0100007447.hg.1 | Centrosomal protein 85kda | CEP85 | -1.52 | 2.97E-02 | -2.83 | 7.82E-05 |
| TC1200010653.hg.1 | Ceramide synthase 5 | CERS5 | -2.09 | 1.40E-03 | -1.95 | 2.70E-03 |
| TC0300010357.hg.1 | Coiled-coil-helix-coiled-coil-helix domain containing 4 | CHCHD4 | -1.56 | 2.37E-02 | -1.91 | 3.00E-03 |
| TC1900011635.hg.1 | Charged multivesicular body protein 2A | CHMP2A | -1.53 | 2.03E-02 | -3.01 | 2.45E-05 |
| TC1700010747.hg.1 | Cytochrome c oxidase assembly factor 3 | COA3 | -1.77 | 7.30E-03 | -1.72 | 9.60E-03 |
| TC1800007850.hg.1 | Collectin sub-family member 12 | COLEC12 | -1.53 | 4.48E-02 | -1.77 | 1.15E-02 |
| TC1200007759.hg.1 | Coatomer protein complex subunit zeta 1 | COPZ1 | -1.6 | 9.40E-03 | -1.68 | 5.30E-03 |
| TC1100011829.hg.1 | CREB/ATF bzip transcription factor | CREBZF | -1.61 | 1.13E-02 | -2.35 | 2.00E-04 |
| TC0X00007923.hg.1 | Cleavage stimulation factor, 3 pre-RNA, subunit 2 | CSTF2 | -1.67 | 8.80E-03 | -1.93 | 1.90E-03 |
| TC0100018442.hg.1 | Cytochrome b5 reductase-like | CYB5RL | -1.71 | 1.23E-02 | -1.58 | 2.68E-02 |
| TC0100010661.hg.1 | Aspartyl-trna synthetase 2, mitochondrial | DARS2 | -1.6 | 1.02E-02 | -1.88 | 1.70E-03 |
| TC1200012629.hg.1 | DAZ associated protein 2 | DAZAP2 | -1.68 | 6.90E-03 | -1.81 | 3.10E-03 |
| TC1600007234.hg.1 | Dynactin 5 (p25) | DCTN5 | -1.62 | 2.15E-02 | -1.59 | 2.69E-02 |
| TC1700012089.hg.1 | Dicarbonyl/L-xylulose reductase | DCXR | -1.55 | 3.81E-02 | -2.08 | 2.40E-03 |
| TC0100006627.hg.1 | DNA fragmentation factor, 40kda, beta polypeptide (caspase-activated dnase) | DFFB | -1.5 | 3.56E-02 | -1.67 | 1.16E-02 |
| TC1700010221.hg.1 | Dehydrogenase/reductase (SDR family) member 13 | DHRS13 | -1.82 | 4.10E-02 | -1.95 | 2.56E-02 |
| TC0200012299.hg.1 | DEAH (Asp-Glu-Ala-Asp/His) box polypeptide 57 | DHX57 | -1.65 | 3.44E-02 | -1.98 | 7.20E-03 |
| TC1100009043.hg.1 | DIX domain containing 1 | DIXDC1 | -1.8 | 2.51E-02 | -1.89 | 1.73E-02 |
| TC1700010701.hg.1 | Dnaj (Hsp40) homolog, subfamily C, member 7 | DNAJC7 | -1.74 | 2.48E-02 | -3.29 | 2.00E-04 |
| TC1700010700.hg.1 | Zhang2013 ALT_ACCEPTOR, ALT_DONOR, coding, INTERNAL, intronic, OVERLAPTX, OVEXON best transcript NM_003315 | DNAJC7 | -2.08 | 2.40E-03 | -2.39 | 7.00E-04 |
| TC0200015779.hg.1 | Aspartyl aminopeptidase | DNPEP | -1.51 | 3.28E-02 | -1.9 | 3.10E-03 |
| TC2000007502.hg.1 | Deoxynucleotidyltransferase, terminal, interacting protein 1 | DNTTIP1 | -1.51 | 3.04E-02 | -1.62 | 1.44E-02 |
| TC1100009419.hg.1 | Etoposide induced 2.4 | EI24 | -1.75 | 4.10E-03 | -2.1 | 6.00E-04 |
| TC1500006930.hg.1 | Eukaryotic translation initiation factor 2 alpha kinase 4 | EIF2AK4 | -1.56 | 2.14E-02 | -2.39 | 3.00E-04 |
| TC1200010038.hg.1 | Epidermal growth factor receptor pathway substrate 8 | EPS8 | -1.67 | 1.02E-02 | -1.57 | 1.88E-02 |
| TC0400009132.hg.1 | Electron-transferring-flavoprotein dehydrogenase | ETFDH | -1.55 | 4.25E-02 | -2.03 | 3.50E-03 |
| TC0500009803.hg.1 | Memczak2013 ANTISENSE, CDS, coding, INTERNAL best transcript NM_007277 | EXOC3 | -1.66 | 2.16E-02 | -1.71 | 4.46E-02 |
| TC0900008967.hg.1 | Exosome component 2 | EXOSC2 | -1.66 | 4.90E-03 | -1.57 | 9.70E-03 |
| TC1600006493.hg.1 | Family with sequence similarity 173, member A | FAM173A | -1.54 | 3.84E-02 | -1.73 | 1.20E-02 |
| TC0X00008833.hg.1 | Family with sequence similarity 50, member A | FAM50A | -1.65 | 4.26E-02 | -1.89 | 1.37E-02 |
| TC0600009102.hg.1 | FIG4 phosphoinositide 5-phosphatase | FIG4 | -2.15 | 1.90E-03 | -1.66 | 2.06E-02 |
| TC0700010046.hg.1 | Ftsj RNA methyltransferase homolog 2 (E. Coli) | FTSJ2 | -1.88 | 9.40E-03 | -2.19 | 2.60E-03 |
| TC0800007985.hg.1 | Ganglioside induced differentiation associated protein 1 | GDAP1 | -1.66 | 3.79E-02 | -1.71 | 2.98E-02 |
| TC0500012568.hg.1 | Gem nuclear organelle associated protein 5 | GEMIN5 | -2.1 | 6.00E-04 | -1.53 | 2.05E-02 |
| TC2000007016.hg.1 | GINS complex subunit 1 (Psf1 homolog) | GINS1 | -1.9 | 1.10E-03 | -1.57 | 1.06E-02 |
| TC1400010632.hg.1 | G-patch domain containing 2 like | GPATCH2L | -1.54 | 1.45E-02 | -1.88 | 1.50E-03 |
| TC0100012788.hg.1 | G protein-coupled receptor 157 | GPR157 | -2.09 | 2.90E-03 | -2.33 | 1.10E-03 |
| TC1600007723.hg.1 | Glutamic pyruvate transaminase (alanine aminotransferase) 2 | GPT2 | -1.61 | 3.20E-02 | -1.9 | 6.80E-03 |
| TC1700006592.hg.1 | Germ cell associated 2 (haspin) | GSG2 | -1.62 | 1.69E-02 | -2.53 | 2.00E-04 |
| TC0100013998.hg.1 | HECT domain containing E3 ubiquitin protein ligase 3 | HECTD3 | -1.61 | 1.38E-02 | -2.07 | 1.00E-03 |
| TC0100009877.hg.1 | Histone cluster 2, h3a | HIST2H3A | -3.84 | 9.82E-06 | -2.76 | 1.00E-04 |
| TC0100015701.hg.1 | Histone cluster 2, h3a; histone cluster 2, h3c | HIST2H3A; HIST2H3C | -4.41 | 9.01E-06 | -3.01 | 1.00E-04 |
| TC0100015707.hg.1 | Histone cluster 2, h4a; histone cluster 2, h4b | HIST2H4A; HIST2H4B | -3.38 | 7.44E-05 | -2.77 | 3.00E-04 |
| TC0100009870.hg.1 | Histone cluster 2, h4b; histone cluster 2, h4a | HIST2H4B; HIST2H4A | -3.27 | 2.28E-05 | -2.92 | 5.53E-05 |
| TC0700010563.hg.1 | Homeobox A7 | HOXA7 | -2.44 | 2.00E-04 | -1.65 | 1.08E-02 |
| TC1600011047.hg.1 | Hydroxysteroid dehydrogenase like 1 | HSDL1 | -1.64 | 3.57E-02 | -1.64 | 3.45E-02 |
| TC1700008879.hg.1 | Immature colon carcinoma transcript 1 | ICT1 | -1.67 | 2.08E-02 | -1.75 | 1.35E-02 |
| TC1300008713.hg.1 | Kelch repeat and BTB (POZ) domain containing 6 | KBTBD6 | -2.01 | 8.00E-03 | -3.26 | 2.00E-04 |
| TC0600007836.hg.1 | Potassium channel tetramerization domain containing 20 | KCTD20 | -1.71 | 4.20E-03 | -2.08 | 5.00E-04 |
| TC0100016476.hg.1 | Kiaa0040 | KIAA0040 | -1.73 | 7.70E-03 | -1.52 | 3.09E-02 |
| TC1600009646.hg.1 | Lysine-rich nucleolar protein 1 | KNOP1 | -1.7 | 1.16E-02 | -1.53 | 3.47E-02 |
| TC2100007362.hg.1 | Leucine rich repeat containing 3 | LRRC3 | -1.51 | 4.66E-02 | -1.76 | 1.07E-02 |
| TC1900010011.hg.1 | LSM4 homolog, U6 small nuclear RNA and mrna degradation associated | LSM4 | -1.5 | 1.63E-02 | -1.71 | 3.30E-03 |
| TC0600011808.hg.1 | Mediator complex subunit 20 | MED20 | -1.78 | 8.50E-03 | -2.61 | 3.00E-04 |
| TC1000011659.hg.1 | Meningioma expressed antigen 5 (hyaluronidase) | MGEA5 | -1.57 | 3.37E-02 | -1.64 | 7.80E-03 |
| TC0300006631.hg.1 | Makorin ring finger protein 2 | MKRN2 | -1.68 | 6.70E-03 | -1.8 | 3.20E-03 |
| TC0100008124.hg.1 | Methylmalonic aciduria (cobalamin deficiency) cblc type, with homocystinuria | MMACHC | -1.81 | 7.80E-03 | -1.82 | 7.30E-03 |
| TC1000011555.hg.1 | MORN repeat containing 4 | MORN4 | -1.69 | 1.05E-02 | -2.51 | 2.00E-04 |
| TC1500007860.hg.1 | Mannose phosphate isomerase | MPI | -1.96 | 1.30E-03 | -2.02 | 1.00E-03 |
| TC1900008036.hg.1 | Mitochondrial ribosomal protein S12 | MRPS12 | -1.79 | 2.95E-02 | -1.99 | 1.32E-02 |
| TC1600009063.hg.1 | Mitochondrial ribosomal protein S34 | MRPS34 | -1.66 | 9.20E-03 | -1.68 | 7.80E-03 |
| TC0500011255.hg.1 | Metaxin 3 | MTX3 | -1.79 | 1.50E-02 | -1.67 | 2.76E-02 |
| TC0400012933.hg.1 | N-acylethanolamine acid amidase | NAAA | -1.64 | 7.30E-03 | -1.66 | 6.40E-03 |
| TC0300013727.hg.1 | Nuclear cap binding protein subunit 2 | NCBP2 | -1.62 | 2.73E-02 | -1.62 | 2.68E-02 |
| TC1000008961.hg.1 | NHL repeat containing 2 | NHLRC2 | -1.97 | 6.00E-04 | -1.52 | 1.38E-02 |
| TC0500012937.hg.1 | NOP16 nucleolar protein | NOP16 | -1.88 | 3.10E-03 | -2.24 | 5.00E-04 |
| TC0600014283.hg.1 | Nudix hydrolase 3 | NUDT3 | -1.59 | 1.17E-02 | -1.74 | 4.30E-03 |
| TC0X00009929.hg.1 | Oligophrenin 1 | OPHN1 | -1.69 | 3.18E-02 | -1.64 | 3.94E-02 |
| TC0500010680.hg.1 | Memczak2013 ANTISENSE, CDS, coding, INTERNAL, intronic best transcript NM_001178056 | PARP8 | -1.71 | 1.82E-02 | -3.63 | 4.02E-05 |
| TC1100009700.hg.1 | Parkinson disease 7 domain containing 1 | PDDC1 | -1.72 | 2.33E-02 | -1.85 | 1.25E-02 |
| TC1000008574.hg.1 | Phosphoglycerate mutase 1 (brain) | PGAM1 | -1.51 | 3.00E-02 | -2.07 | 1.10E-03 |
| TC1700009623.hg.1 | PHD finger protein 23 | PHF23 | -2.01 | 8.00E-04 | -1.6 | 9.90E-03 |
| TC1900006956.hg.1 | Peptidylprolyl cis/trans isomerase, NIMA-interacting 1 | PIN1 | -1.56 | 4.91E-02 | -2.59 | 7.00E-04 |
| TC1100010216.hg.1 | Pleckstrin homology domain containing, family A member 7 | PLEKHA7 | -1.72 | 4.70E-02 | -3.23 | 6.00E-04 |
| TC0100015931.hg.1 | Phosphomevalonate kinase | PMVK | -1.6 | 2.80E-02 | -2.21 | 1.40E-03 |
| TC0600008095.hg.1 | Polymerase (RNA) I polypeptide C | POLR1C | -1.84 | 4.40E-03 | -1.6 | 1.89E-02 |
| TC1000008193.hg.1 | Peptidylprolyl isomerase F | PPIF | -1.51 | 2.89E-02 | -2.97 | 4.02E-05 |
| TC0900008902.hg.1 | Protein phosphatase 2A activator, regulatory subunit 4 | PPP2R4 | -1.54 | 1.54E-02 | -2.4 | 1.00E-04 |
| TC0100008450.hg.1 | Protein kinase, AMP-activated, alpha 2 catalytic subunit | PRKAA2 | -1.65 | 2.98E-02 | -1.73 | 1.95E-02 |
| TC1100012717.hg.1 | Pseudouridylate synthase 3 | PUS3 | -1.57 | 2.41E-02 | -1.67 | 1.29E-02 |
| TC0400012905.hg.1 | Quinoid dihydropteridine reductase | QDPR | -1.64 | 1.74E-02 | -2.17 | 1.10E-03 |
| TC1500010756.hg.1 | RAB11A, member RAS oncogene family | RAB11A | -1.57 | 2.73E-02 | -1.63 | 1.79E-02 |
| TC0800010138.hg.1 | RAB11 family interacting protein 1 (class I) | RAB11FIP1 | -1.5 | 4.02E-02 | -1.81 | 6.10E-03 |
| TC1400009422.hg.1 | RAB15, member RAS oncogene family | RAB15 | -1.62 | 1.94E-02 | -1.75 | 9.00E-03 |
| TC0X00008405.hg.1 | RNA binding motif protein, X-linked 2 | RBMX2 | -1.55 | 1.89E-02 | -1.76 | 4.90E-03 |
| TC0200016618.hg.1 | Ribonuclease H1 | RNASEH1 | -2.08 | 8.00E-04 | -1.57 | 1.65E-02 |
| TC1800009222.hg.1 | Ring finger protein 125, E3 ubiquitin protein ligase | RNF125 | -1.83 | 2.80E-02 | -1.69 | 4.96E-02 |
| TC1200010901.hg.1 | Ring finger protein 41, E3 ubiquitin protein ligase | RNF41 | -1.71 | 3.20E-03 | -1.65 | 4.70E-03 |
| TC0200008351.hg.1 | Ribose 5-phosphate isomerase A | RPIA | -1.66 | 3.35E-02 | -2.48 | 1.20E-03 |
| TC0600014095.hg.1 | Ribonuclease P/MRP 21kda subunit | RPP21 | -1.93 | 1.40E-02 | -1.85 | 1.95E-02 |
| TC1700012287.hg.1 | Ribosomal protein S6 kinase, 70kda, polypeptide 1 | RPS6KB1 | -1.57 | 2.86E-02 | -1.54 | 3.36E-02 |
| TC0600014130.hg.1 | Ribosomal RNA processing 36 | RRP36 | -2.33 | 8.00E-04 | -1.59 | 2.79E-02 |
| TC1900009805.hg.1 | Sterile alpha motif domain containing 1 | SAMD1 | -1.6 | 4.04E-02 | -2.26 | 2.00E-03 |
| TC0800012165.hg.1 | Scribbled planar cell polarity protein; microrna 937 | SCRIB; MIR937 | -1.6 | 8.80E-03 | -2.44 | 9.82E-05 |
| TC1400008760.hg.1 | Short chain dehydrogenase/reductase family 39U, member 1 | SDR39U1 | -1.59 | 1.18E-02 | -1.79 | 3.00E-03 |
| TC0100013760.hg.1 | Splicing factor 3a subunit 3 | SF3A3 | -1.72 | 9.30E-03 | -1.97 | 2.20E-03 |
| TC1400009351.hg.1 | SIX homeobox 1 | SIX1 | -1.54 | 2.15E-02 | -1.74 | 5.40E-03 |
| TC1700010826.hg.1 | Solute carrier family 25, member 39 | SLC25A39 | -1.56 | 2.61E-02 | -1.51 | 3.65E-02 |
| TC0100007954.hg.1 | Small arfgap2 | SMAP2 | -1.73 | 5.10E-03 | -1.63 | 9.70E-03 |
| TC1700012401.hg.1 | SWI/SNF related, matrix associated, actin dependent regulator of chromatin, subfamily e, member 1 | SMARCE1 | -1.83 | 4.30E-03 | -1.57 | 2.11E-02 |
| TC1700012286.hg.1 | SMG8 nonsense mediated mrna decay factor | SMG8 | -2.32 | 1.20E-03 | -1.58 | 3.66E-02 |
| TC0100018430.hg.1 | Small integral membrane protein 12 | SMIM12 | -1.56 | 1.59E-02 | -1.75 | 4.30E-03 |
| TC0100011963.hg.1 | Sprt-like N-terminal domain | SPRTN | -1.58 | 2.48E-02 | -1.94 | 3.40E-03 |
| TC1200007842.hg.1 | SPRY domain containing 4 | SPRYD4 | -1.81 | 2.60E-03 | -2.52 | 8.65E-05 |
| TC1400006890.hg.1 | Signal recognition particle 54kda | SRP54 | -1.53 | 4.23E-02 | -1.66 | 1.98E-02 |
| TC1700012437.hg.1 | Serine/arginine-rich splicing factor 1 | SRSF1 | -1.59 | 1.35E-02 | -1.82 | 3.00E-03 |
| TC0100013323.hg.1 | Sperm-tail PG-rich repeat containing 1 | STPG1 | -1.83 | 1.80E-03 | -1.61 | 8.10E-03 |
| TC1600011428.hg.1 | Telomeric repeat binding factor 2, interacting protein | TERF2IP | -1.59 | 8.60E-03 | -1.96 | 8.00E-04 |
| TC0300008477.hg.1 | Translocase of inner mitochondrial membrane domain containing 1 | TIMMDC1 | -1.58 | 1.54E-02 | -1.94 | 1.60E-03 |
| TC1400010723.hg.1 | Transmembrane 9 superfamily member 1 | TM9SF1 | -1.54 | 4.25E-02 | -1.69 | 1.84E-02 |
| TC0700010926.hg.1 | Transmembrane p24 trafficking protein 4 | TMED4 | -1.76 | 1.27E-02 | -1.7 | 1.76E-02 |
| TC0200016516.hg.1 | Transmembrane protein 177 | TMEM177 | -1.59 | 2.89E-02 | -1.54 | 3.93E-02 |
| TC1900011867.hg.1 | Transmembrane protein 205 | TMEM205 | -1.5 | 3.74E-02 | -2.22 | 7.00E-04 |
| TC1700007360.hg.1 | Transmembrane protein 97 | TMEM97 | -1.63 | 1.74E-02 | -1.85 | 4.80E-03 |
| TC0800006681.hg.1 | Tankyrase, TRF1-interacting ankyrin-related ADP-ribose polymerase | TNKS | -1.66 | 2.17E-02 | -2.57 | 4.00E-04 |
| TC2200007361.hg.1 | Translocase of outer mitochondrial membrane 22 homolog (yeast) | TOMM22 | -1.78 | 3.20E-03 | -1.56 | 1.38E-02 |
| TC1700012352.hg.1 | Trafficking protein particle complex 1 | TRAPPC1 | -1.51 | 1.61E-02 | -2.16 | 3.00E-04 |
| TSUnmapped00000493.hg.1 | Trafficking protein particle complex 4 | TRAPPC4 | -1.51 | 2.13E-02 | -1.6 | 2.87E-02 |
| TC0900008574.hg.1 | Tripartite motif containing 32 | TRIM32 | -1.75 | 3.77E-02 | -3.12 | 6.00E-04 |
| TC2200008018.hg.1 | Trna methyltransferase 2 homolog A | TRMT2A | -1.57 | 1.43E-02 | -1.58 | 1.29E-02 |
| TC0600012958.hg.1 | TSPY-like 4 | TSPYL4 | -2.23 | 6.00E-04 | -2.55 | 2.00E-04 |
| TC0900010946.hg.1 | Thiosulfate sulfurtransferase (rhodanese)-like domain containing 2 | TSTD2 | -1.75 | 5.10E-03 | -2.34 | 3.00E-04 |
| TC2000009053.hg.1 | TELO2 interacting protein 1 | TTI1 | -1.77 | 4.60E-03 | -1.74 | 5.30E-03 |
| TC1800006731.hg.1 | Tubulin, beta 6 class V | TUBB6 | -2.13 | 1.50E-03 | -1.85 | 5.70E-03 |
| TC1300008080.hg.1 | Memczak2013 ANTISENSE, coding, INTERNAL, intronic best transcript NM_006322 | TUBGCP3 | -2.01 | 5.30E-03 | -1.95 | 6.70E-03 |
| TC1100012358.hg.1 | Ubiquitin specific peptidase 28 | USP28 | -1.76 | 1.62E-02 | -2.1 | 3.50E-03 |
| TC0800008626.hg.1 | UTP23, small subunit (SSU) processome component, homolog (yeast) | UTP23 | -1.64 | 1.24E-02 | -1.86 | 3.30E-03 |
| TC1400009784.hg.1 | VPS33B interacting protein, apical-basolateral polarity regulator, spe-39 homolog | VIPAS39 | -1.52 | 4.00E-02 | -1.58 | 2.74E-02 |
| TC1400010764.hg.1 | Vesicle transport through interaction with t-snares 1B | VTI1B | -1.59 | 2.69E-02 | -2.03 | 2.50E-03 |
| TC0100009580.hg.1 | WD repeat domain 3 | WDR3 | -1.52 | 3.55E-02 | -2.54 | 3.00E-04 |
| TC0900011650.hg.1 | WD repeat domain 34 | WDR34 | -1.96 | 1.30E-02 | -1.68 | 4.44E-02 |
| TC0200016681.hg.1 | WD repeat domain 92 | WDR92 | -1.55 | 1.60E-02 | -1.94 | 1.30E-03 |
| TC0600006611.hg.1 | Werner helicase interacting protein 1 | WRNIP1 | -1.7 | 3.52E-02 | -1.75 | 2.84E-02 |
| TC1200010944.hg.1 | Zinc finger and BTB domain containing 39 | ZBTB39 | -1.9 | 2.20E-03 | -3.15 | 2.22E-05 |
| TC0600014112.hg.1 | Zinc finger and BTB domain containing 9 | ZBTB9 | -1.98 | 8.60E-03 | -1.73 | 2.59E-02 |
| TC1900011935.hg.1 | ZFP30 zinc finger protein | ZFP30 | -1.72 | 1.84E-02 | -1.6 | 3.51E-02 |
| TC1900011814.hg.1 | Zinc finger protein 17 | ZNF17 | -1.66 | 4.67E-02 | -1.88 | 1.74E-02 |
| TC1900008655.hg.1 | Zinc finger protein 175 | ZNF175 | -2.19 | 5.00E-04 | -2.66 | 8.46E-05 |
| TC1900011752.hg.1 | Zinc finger protein 223 | ZNF223 | -1.59 | 4.03E-02 | -1.58 | 4.43E-02 |
| TC1700009551.hg.1 | Zinc finger protein 232 | ZNF232 | -2.16 | 8.20E-03 | -2.57 | 2.30E-03 |
| TC1200012737.hg.1 | Zinc finger protein 268 | ZNF268 | -1.57 | 2.33E-02 | -1.61 | 1.79E-02 |
| TC2000009973.hg.1 | Zinc finger protein 343 | ZNF343 | -1.57 | 3.27E-02 | -2.79 | 2.00E-04 |
| TSUnmapped00000461.hg.1 | Zinc finger protein 35 | ZNF35 | -2.34 | 7.00E-04 | -1.68 | 1.57E-02 |
| TSUnmapped00000018.hg.1 | Zinc finger protein 35 | ZNF35 | -2.58 | 1.00E-04 | -1.53 | 2.63E-02 |
| TC0600014086.hg.1 | Zinc finger protein 391 | ZNF391 | -1.61 | 2.02E-02 | -1.94 | 3.10E-03 |
| TC0200016418.hg.1 | Zinc finger protein 512 | ZNF512 | -1.9 | 2.40E-03 | -1.72 | 7.00E-03 |
| TC1900009002.hg.1 | Zinc finger protein 530 | ZNF530 | -1.8 | 1.41E-02 | -1.68 | 2.61E-02 |
| TSUnmapped00000068.hg.1 | Zinc finger protein 780A | ZNF780A | -2.23 | 9.50E-03 | -2.01 | 1.60E-03 |
| TSUnmapped00000038.hg.1 | Zinc finger protein 780A | ZNF780A | -1.84 | 3.80E-03 | -2.08 | 1.54E-02 |
| TC2000007507.hg.1 | Zinc finger, SWIM-type containing 1 | ZSWIM1 | -1.6 | 3.17E-02 | -1.94 | 5.60E-03 |

**Table S4.** Upregulated (A) and downregulated (B) genes identified with RNA sequencing after 4 hours exposition to exogenous MATLND4 in HEK-293T cells cultured in DMEM low glucose.

| A. Upregulated genes | | | | | |
| --- | --- | --- | --- | --- | --- |
| Ensembl ID | Gene symbol | Description | Group | Fold Change (logFC) | Adjusted P value |
| ENSG00000231864.2 |  |  | lncRNA | 2,32 | 0,023734 |
| ENSG00000224962.4 | PSAT1P4 | phosphoserine aminotransferase 1 pseudogene 4 | Pseudogene | 1,58 | 0,023734 |
| ENSG00000213608.5 | SLC25A14P1 | solute carrier family 25 member 14 pseudogene 1 | Pseudogene | 1,58 | 0,023734 |
| ENSG00000251739.1 | RNU6-1053P | RNA, U6 small nuclear 1053, pseudogene | Pseudogene | 1,58 | 0,023734 |
| ENSG00000146005.4 | PSD2 | pleckstrin and Sec7 domain containing 2 | Coding | 1,58 | 0,023734 |
| ENSG00000250909.1 |  |  | lncRNA | 1,58 | 0,023734 |
| ENSG00000272168.10 | CASC15 | cancer susceptibility 15 | lncRNA | 1,58 | 0,023734 |
| ENSG00000253663.1 | NPM1P52 | nucleophosmin 1 pseudogene 52 | Pseudogene | 1,58 | 0,023734 |
| ENSG00000291170.1 |  |  | lncRNA | 1,58 | 0,023734 |
| ENSG00000075073.16 | TACR2 | tachykinin receptor 2 | Coding | 1,58 | 0,023734 |
| ENSG00000179715.13 | PCED1B | PC-esterase domain containing 1B | Coding | 1,58 | 0,023734 |
| ENSG00000279122.1 |  |  | TEC | 1,58 | 0,023734 |
| ENSG00000262921.1 |  |  | lncRNA | 1,58 | 0,023734 |
| ENSG00000277249.1 | MIR6784 | microRNA 6784 | miRNA | 1,58 | 0,023734 |
| ENSG00000070731.11 | ST6GALNAC2 | ST6 N-acetylgalactosaminide alpha-2,6-sialyltransferase 2ST6 N-acetylgalactosaminide alpha-2,6-sialyltransferase 2 | Coding | 1,58 | 0,023734 |
| ENSG00000267289.1 | PIN1-DT | PIN1 divergent transcript | lncRNA | 1,58 | 0,023734 |
| ENSG00000184844.6 | CYCSP45 | CYCS pseudogene 45 | Pseudogene | 1,58 | 0,023734 |
| ENSG00000140650.13 | PMM2 | phosphomannomutase 2 | Coding | 0,20 | 0,047526 |
| B. Downregulated genes | | | | | |
| ENSG00000227183.3 | HDGFP1 | heparin binding growth factor pseudogene 1 | Pseudogene | -2,64 | 0,043903 |
| ENSG00000276840.1 | PMS2P13 | PMS1 homolog 2, mismatch repair system component pseudogene 13 | Pseudogene | -2,32 | 0,023734 |
| ENSG00000261359.2 | PYCARD-AS1 | PYCARD antisense RNA 1 | lncRNA | -2,32 | 0,023734 |
| ENSG00000138100.14 | TRIM54 | tripartite motif containing 54 | Coding | -1,59 | 0,023734 |
| ENSG00000163060.8 | TEKT4 | tektin 4 | Coding | -1,59 | 0,023734 |
| ENSG00000214147.2 | ENAHP1 | ENAH pseudogene 1 | Pseudogene | -1,59 | 0,023734 |
| ENSG00000203801.8 | LINC00222 | long intergenic non-protein coding RNA 222 | lncRNA | -1,59 | 0,023734 |
| ENSG00000164744.14 | SUN3 | Sad1 and UNC84 domain containing 3 | Coding | -1,59 | 0,023734 |
| ENSG00000237749.4 | RPS27P9 | ribosomal protein S27 pseudogene 9 | Pseudogene | -1,59 | 0,023734 |
| ENSG00000229399.1 | PFDN5 | prefoldin 5 (PFDN5) pseudogene | Pseudogene | -1,59 | 0,023734 |
| ENSG00000224858.5 | RPL29P11 | ribosomal protein L29 pseudogene 11 | Pseudogene | -1,59 | 0,023734 |
| ENSG00000278974.1 |  |  | lncRNA | -1,59 | 0,023734 |
| ENSG00000010030.14 | ETV7 | ETS variant transcription factor 7 | Coding | -1,59 | 0,023734 |
| ENSG00000273819.1 | ENPP7P7 | ectonucleotide pyrophosphatase/phosphodiesterase 7 pseudogene 7 | Pseudogene | -1,59 | 0,023734 |
| ENSG00000275936.1 |  |  | lncRNA | -1,59 | 0,023734 |
| ENSG00000248079.4 | DPH6-DT | DPH6 divergent transcript | lncRNA | -1,59 | 0,023734 |
| ENSG00000260468.1 | LINC01290 | long intergenic non-protein coding RNA 1290 | lncRNA | -1,59 | 0,023734 |
| ENSG00000288909.2 |  |  | lncRNA | -1,59 | 0,023734 |
| ENSG00000141934.10 | PLPP2 | phospholipid phosphatase 2 | Coding | -1,59 | 0,023734 |
| ENSG00000269959.1 | SPACA6-AS1 | SPACA6 antisense RNA 1 | lncRNA | -1,59 | 0,023734 |
| ENSG00000196933.5 | RPS26P11 | ribosomal protein S26 pseudogene 11 | Pseudogene | -1,59 | 0,023734 |
| ENSG00000225912.1 | RPL26P36 | ribosomal protein L26 pseudogene 36 | Pseudogene | -1,59 | 0,023734 |

**Table S5**. Upregulated (A) and downregulated (B) genes identified with RNA sequencing after 72 hours exposition to exogenous MATLND4 in HEK-293T cells cultured in DMEM low glucose.

| A. Upregulated genes | | | | | |
| --- | --- | --- | --- | --- | --- |
| Ensembl ID | Gene symbol | Description | Group | Fold Change (logFC) | Adjusted P value |
| ENSG00000260896.6 | ARLNC1 | Androgen Receptor Regulated Long Noncoding RNA 1 | lncRNA | 2,69 | 0,025782 |
| ENSG00000258982.1 |  |  | lncRNA | 2,54 | 0,022146 |
| ENSG00000260179.1 |  |  | lncRNA | 2,37 | 0,022146 |
| ENSG00000162494.6 | LRRC38 | leucine rich repeat containing 38 | Coding | 2,37 | 0,022146 |
| ENSG00000225423.1 | TNPO1P1 | transportin 1 pseudogene 1 | Pseudogene | 2,37 | 0,022146 |
| ENSG00000188060.8 | RAB42 | RAB42, member RAS oncogene family | Coding | 1,64 | 0,022146 |
| ENSG00000229431.1 | MED8-AS1 | MED8 Antisense RNA 1 | lncRNA | 1,64 | 0,022146 |
| ENSG00000291326.1 |  |  | lncRNA | 1,64 | 0,022146 |
| ENSG00000159176.14 | CSRP1 | cysteine and glycine rich protein 1 | Coding | 1,64 | 0,022146 |
| ENSG00000196878.15 | LAMB3 | laminin subunit beta 3 | Coding | 1,64 | 0,022146 |
| ENSG00000287291.1 |  |  | lncRNA | 1,64 | 0,022146 |
| ENSG00000286707.1 |  |  | lncRNA | 1,64 | 0,022146 |
| ENSG00000157856.12 | DRC1 | dynein regulatory complex subunit 1 | Coding | 1,64 | 0,022146 |
| ENSG00000288866.1 |  |  | lncRNA | 1,64 | 0,022146 |
| ENSG00000286942.1 |  |  | lncRNA | 1,64 | 0,022146 |
| ENSG00000242829.1 | RPS26P21 | ribosomal protein S26 pseudogene 21 | Pseudogene | 1,64 | 0,022146 |
| ENSG00000156194.19 | PPEF2 | protein phosphatase with EF-hand domain 2 | Coding | 1,64 | 0,022146 |
| ENSG00000276945.1 |  |  | Pseudogene | 1,64 | 0,022146 |
| ENSG00000289274.1 |  |  | lncRNA | 1,64 | 0,022146 |
| ENSG00000213316.10 | LTC4S | leukotriene C4 synthase | Coding | 1,64 | 0,022146 |
| ENSG00000236512.1 | RPL29P1 | Ribosomal Protein L29 Pseudogene 1 | Pseudogene | 1,64 | 0,022146 |
| ENSG00000286277.1 |  |  | lncRNA | 1,64 | 0,022146 |
| ENSG00000244710.3 | RN7SL47P | RNA, 7SL, Cytoplasmic 47, Pseudogene | Pseudogene | 1,64 | 0,022146 |
| ENSG00000224273.2 | RABGEF1P3 | RABGEF1 Pseudogene 3 | Pseudogene | 1,64 | 0,022146 |
| ENSG00000167910.4 | CYP7A1 | Cytochrome P450 Family 7 Subfamily A Member 1 | Coding | 1,64 | 0,022146 |
| ENSG00000235619.1 | RPL36AP33 | Ribosomal Protein L36a Pseudogene 33 | Pseudogene | 1,64 | 0,022146 |
| ENSG00000259286.3 |  |  | Pseudogene | 1,64 | 0,022146 |
| ENSG00000284709.1 |  |  | Pseudogene | 1,64 | 0,022146 |
| ENSG00000244582.2 | RPL21P120 | Ribosomal Protein L21 Pseudogene 120 | Pseudogene | 1,64 | 0,022146 |
| ENSG00000278223.1 | MIR6783 | MicroRNA 6783 | miRNA | 1,64 | 0,022146 |
| ENSG00000269578.1 |  |  | lncRNA | 1,64 | 0,022146 |
| ENSG00000284630.1 |  |  | Pseudogene | 1,64 | 0,022146 |
| ENSG00000279927.1 |  |  | lncRNA | 1,64 | 0,022146 |
| ENSG00000229168.4 | RPL19P20 | Ribosomal Protein L19 Pseudogene 20 | Pseudogene | 1,64 | 0,022146 |
| ENSG00000101842.14 | VSIG1 | V-Set And Immunoglobulin Domain Containing 1 | Coding | 1,64 | 0,022146 |
| B. Downregulated genes | | | | | |
| ENSG00000225978.3 | HAR1A | Highly Accelerated Region 1A | lncRNA | -2,43 | 0,022146 |
| ENSG00000134668.13 | SPOCD1 | SPOC domain containing 1 | Coding | -2,27 | 0,022146 |
| ENSG00000214552.4 | COPS8P2 | COP9 Signalosome Subunit 8 Pseudogene 2 | Pseudogene | -2,27 | 0,022146 |
| ENSG00000251359.4 | WWC2-AS2 | WWC2 Antisense RNA 2 | lncRNA | -2,03 | 0,036457 |
| ENSG00000225471.5 | TPT1P15 | TPT1 Pseudogene 15 | Pseudogene | -2,03 | 0,036457 |
| ENSG00000200421.1 | Y_RNA |  | Y RNA | -1,53 | 0,022146 |
| ENSG00000287919.1 |  |  | lncRNA | -1,53 | 0,022146 |
| ENSG00000115386.6 | REG1A | Regenerating Family Member 1 Alpha | Coding | -1,53 | 0,022146 |
| ENSG00000230650.1 |  |  | Pseudogene | -1,53 | 0,022146 |
| ENSG00000138395.17 | CDK15 | Cyclin Dependent Kinase 15 | Coding | -1,53 | 0,022146 |
| ENSG00000224839.1 | RPL12P17 | Ribosomal Protein L12 Pseudogene 17 | Pseudogene | -1,53 | 0,022146 |
| ENSG00000237126.8 |  |  | lncRNA | -1,53 | 0,022146 |
| ENSG00000233806.9 | LINC01237 | Long Intergenic Non-Protein Coding RNA 1237 | lncRNA | -1,53 | 0,022146 |
| ENSG00000244157.1 | EIF4E2P2 | Eukaryotic Translation Initiation Factor 4E Family Member 2 Pseudogene 2 | Pseudogene | -1,53 | 0,022146 |
| ENSG00000244668.1 | SNRPCP3 | Small Nuclear Ribonucleoprotein Polypeptide C Pseudogene 3 | Pseudogene | -1,53 | 0,022146 |
| ENSG00000289206.1 |  |  | lncRNA | -1,53 | 0,022146 |
| ENSG00000255122.1 |  |  | Pseudogene | -1,53 | 0,022146 |
| ENSG00000212743.2 | LINC02656 | Long Intergenic Non-Protein Coding RNA 2656 | lncRNA | -1,53 | 0,022146 |
| ENSG00000262412.1 |  |  | lncRNA | -1,53 | 0,022146 |
| ENSG00000232767.2 | HSPA12A-AS1 | HSPA12A Antisense RNA 1 | lncRNA | -1,53 | 0,022146 |
| ENSG00000130035.9 | GALNT8 | Polypeptide N-Acetylgalactosaminyltransferase 8 | Coding | -1,53 | 0,022146 |
| ENSG00000241749.4 | RPSAP52 | Ribosomal Protein SA Pseudogene 52 | Pseudogene | -1,53 | 0,022146 |
| ENSG00000263535.2 | AK4P1 | Adenylate Kinase 4 Pseudogene 1 | Pseudogene | -1,53 | 0,022146 |
| ENSG00000267033.1 |  |  | lncRNA | -1,53 | 0,022146 |
| ENSG00000256771.5 | ZNF253 | Zinc Finger Protein 253 | Coding | -1,53 | 0,022146 |
| ENSG00000279322.1 |  |  | TEC | -1,53 | 0,022146 |
| ENSG00000223695.1 | MYH9-DT | MYH9 Divergent Transcript | lncRNA | -1,53 | 0,022146 |
| ENSG00000215771.2 | LRRC37A14P | Leucine Rich Repeat Containing 37 Member A14, Pseudogene | Pseudogene | -1,53 | 0,022146 |

**Table S6**. Upregulated (A) and downregulated (B) genes identified with RNA sequencing after 4 hours exposition to exogenous MATLND4 in HeLa cells cultured in DMEM low glucose.

| A. Upregulated genes | | | | | |
| --- | --- | --- | --- | --- | --- |
| Ensembl ID | Gene symbol | Description | Group | Fold Change (logFC) | Adjusted P value |
| ENSG00000237135.1 | DDX10P1 | DEAD-box helicase 10 pseudogene 1 | Pseudogene | 2,62 | 0,049665 |
| ENSG00000214188.9 | ST7-OT4 | ST7 overlapping transcript 4 | lncRNA | 2,29 | 0,049665 |
| ENSG00000255647.3 | CTD-2373J6.2 | uncharacterized LOC731157 | lncRNA | 1,55 | 0,049665 |
| B. Downregulated genes | | | | | |
| ENSG00000236337.1 | FMR1-IT1 | FMR1 intronic transcript 1 | lncRNA | -2,95 | 0,049665 |
| ENSG00000250324.2 | MRPL22P1 | mitochondrial ribosomal protein L22 pseudogene 1 | Pseudogene | -2,68 | 0,049665 |
| ENSG00000225951.1 | ODF2-AS1 | ODF2 antisense RNA 1 | lncRNA | -2,66 | 0,049665 |
| ENSG00000237758.1 | BANF1P3 | barrier to autointegration factor 1 pseudogene 3 | Pseudogene | -2,34 | 0,049665 |
| ENSG00000288736.2 |  |  | lncRNA | -1,61 | 0,049665 |
| ENSG00000279333.1 |  |  | Uncategorized | -1,61 | 0,049665 |
| ENSG00000237953.1 | RPS13P5 | ribosomal protein S13 (RPS13) pseudogene | Pseudogene | -1,61 | 0,049665 |
| ENSG00000226972.2 | RPL12P19 | ribosomal protein L12 pseudogene 19 | Pseudogene | -1,61 | 0,049665 |
| ENSG00000237260.1 | PPIAP67 | peptidylprolyl Isomerase A Pseudogene 67 | Pseudogene | -1,61 | 0,049665 |
| ENSG00000279320.1 |  |  | TEC | -1,61 | 0,049665 |
| ENSG00000113494.17 | PRLR | Prolactin Receptor | Coding | -1,61 | 0,049665 |
| ENSG00000280420.1 |  |  | TEC | -1,61 | 0,049665 |
| ENSG00000272810.1 |  |  | lncRNA | -1,61 | 0,049665 |
| ENSG00000278959.1 |  |  | TEC | -1,61 | 0,049665 |
| ENSG00000201098.1 | RNY1 | RNA, Ro-associated Y1 | Y RNA | -1,61 | 0,049665 |
| ENSG00000229591.1 |  |  | lncRNA | -1,61 | 0,049665 |
| ENSG00000251191.8 | LINC00589 | Long Intergenic Non-Protein Coding RNA 589 | lncRNA | -1,61 | 0,049665 |
| ENSG00000203321.2 | CARNMT1-AS1 | CARNMT1 Antisense RNA 1 | lncRNA | -1,61 | 0,049665 |
| ENSG00000273262.1 |  | Novel Transcript, Antisense To ACTR1A | lncRNA | -1,61 | 0,049665 |
| ENSG00000008323.16 | PLEKHG6 | Pleckstrin Homology And RhoGEF Domain Containing G6 | Coding | -1,61 | 0,049665 |
| ENSG00000234354.3 | RPS26P47 | Ribosomal Protein S26 Pseudogene 47 | Pseudogene | -1,61 | 0,049665 |
| ENSG00000240624.1 | RPL17P4 | Ribosomal Protein L17 Pseudogene 4 | Pseudogene | -1,61 | 0,049665 |
| ENSG00000254793.1 | FDPSP4 | Farnesyl Diphosphate Synthase Pseudogene 4 | Pseudogene | -1,61 | 0,049665 |
| ENSG00000225798.1 |  |  | lncRNA | -1,61 | 0,049665 |
| ENSG00000253030.1 | MIR2116 | MicroRNA 2116 | miRNA | -1,61 | 0,049665 |
| ENSG00000166104.16 |  | Golgin Subfamily A Pseudogene | Pseudogene | -1,61 | 0,049665 |
| ENSG00000252456.1 | RNA5SP434 | RNA, 5S Ribosomal Pseudogene 434 | Pseudogene | -1,61 | 0,049665 |
| ENSG00000266865.9 | LOC107984974 | SMAD Specific E3 Ubiquitin Protein Ligase 2 (SMURF2) Pseudogene | Pseudogene | -1,61 | 0,049665 |
| ENSG00000267282.1 |  | Novel Transcript, Antisense To PVRL2 | lncRNA | -1,61 | 0,049665 |
| ENSG00000201221.1 | RNU4-40P | RNA, U4 Small Nuclear 40, Pseudogene | Pseudogene | -1,61 | 0,049665 |
| ENSG00000224003.1 | YES1P1 | YES1 Pseudogene 1 | Pseudogene | -1,61 | 0,049665 |
| ENSG00000271886.1 | MIR98 | MicroRNA 98 | miRNA | -1,61 | 0,049665 |
| ENSG00000254791.1 | FAR1-IT1 | FAR1 intronic transcript 1 | lncRNA | -1,49 | 0,049665 |

**Table S7.** Common Gene Ontology results from microarray data on HeLa and HEK-293T cells treated with exogenous MTALTND4 (10 μM) for 4 hours.
